# Supplementary material for: Measurement of the Drug Sensitivity of Single Prostate Cancer Cells
Source: Cancers (Basel). 2021 Dec 2;13(23):6083. doi: 10.3390/cancers13236083 (PMC8656582; doi:10.3390/cancers13236083)
Supplement: Supplementary file 1 [file cancers-13-06083-s001.zip › cancers-1431582-supplementary.pdf]

Supplementary figures:

Measurement of Drug Sensitivity of Single Prostate Cancer Cells

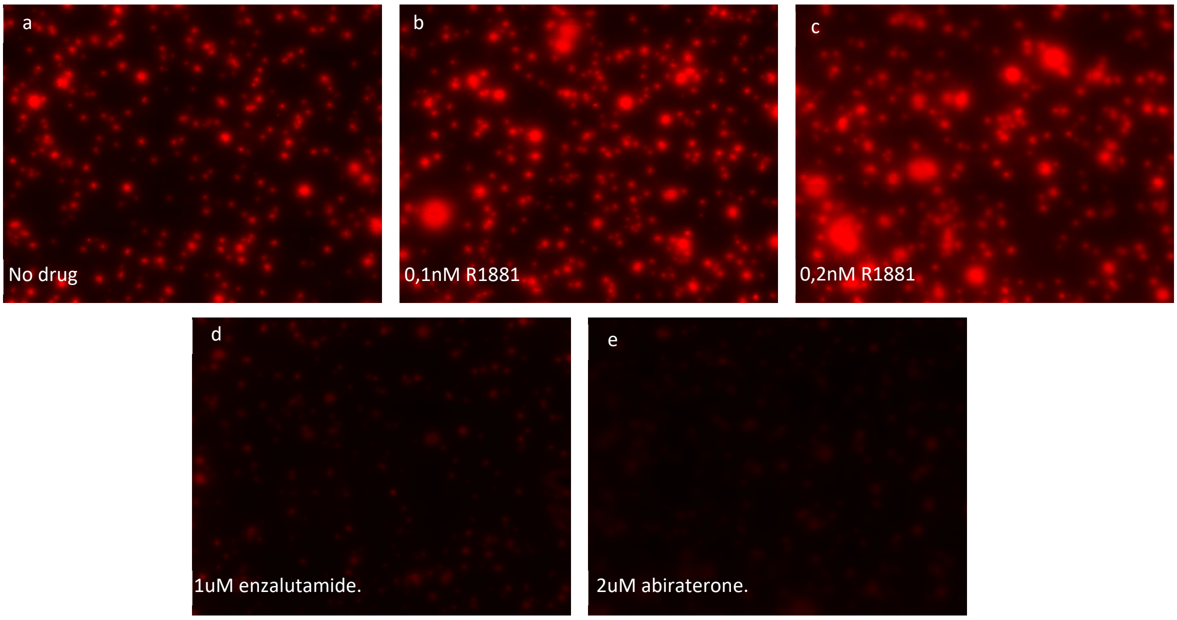

Figures S1: Typical fluorospot images illustrating the secretion of PSA onto PVDF membranes and the effect of drug stimulation. LNCaP cells were seeded onto anti-PSA coated membranes for 24 h to allow cells PSA secretion. (**a**) PSA spots from non-stimulated cells (no drug), (**b**) R1881 stimulated cells (0.1 nM), (**c**) R1881 stimulated cells (0.2 nM), 1uM enzalutamide stimulation (**d**) and 2 uM abiraterone stimulation (**e**).

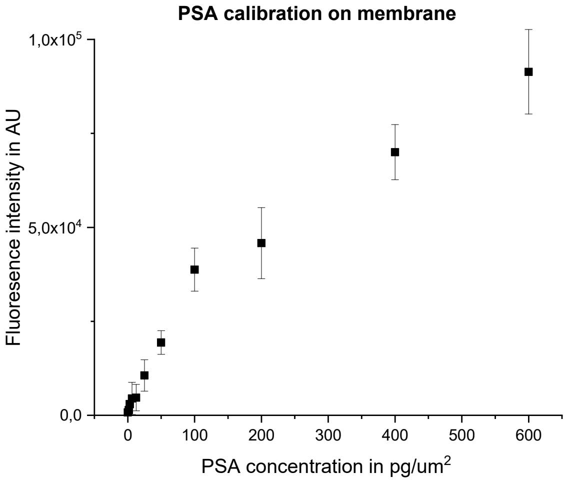

Figure S2. Calibration graphs of fluorescence intensity versus PSA concentration in pg/μm^2 to estimate the amounts of PSA protein secreted by the cells. Cell culture mediums with different known PSA concentrations were spotted as 1 μL drops and fluorescently labelled and detected similar as cell derived PSA membranes. Intensities were quantified and plotted against the concentration in pg/um^2.

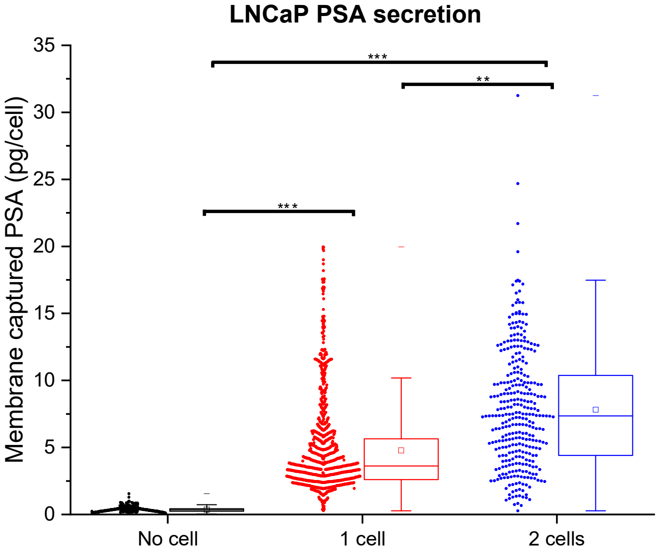

Figure S3. A dot-plot of the PSA secretion from 1 or 2 LNCaP cells in each microwell; each datapoint dot represent the captured amount of PSA at the location of one microwell. The wells that contain 1 cell have significant higher signal than the wells with no cells, and the wells containing two cells show an even higher amount of secreted PSA. Significance levels are indicated with, ** $p \leq 0.001$ and *** $p \leq 0.0001$.

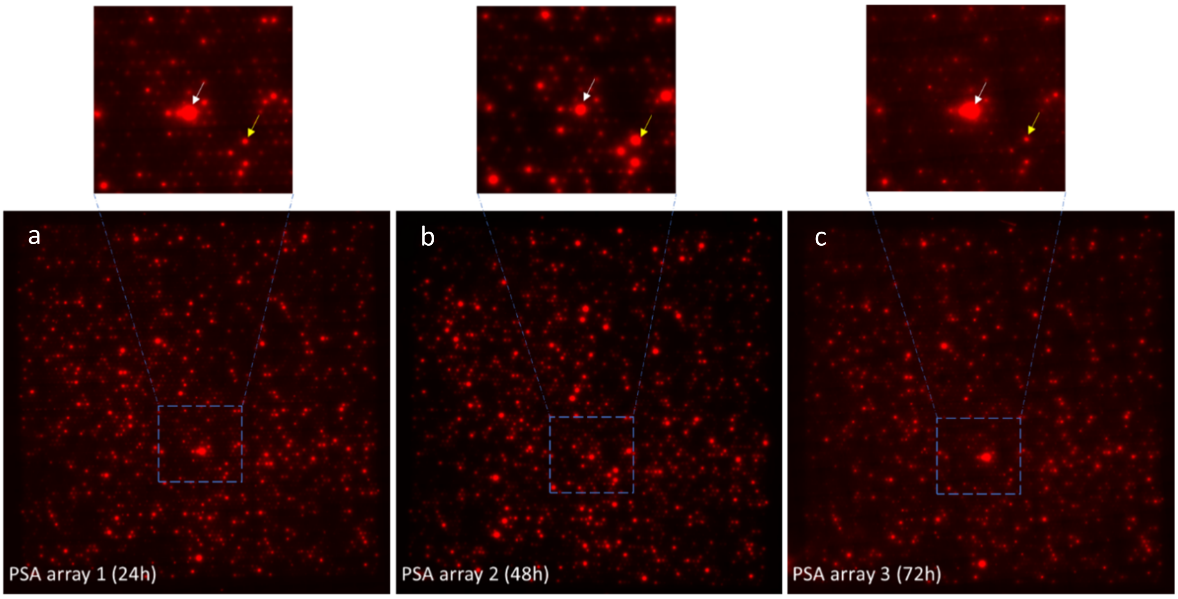

Figure S4. Repeated microwell spotting of cell secreted PSA protein on different PVDF membranes over a 24 h time interval. (**a**) LNCaP cells seeded in the microwell and PSA secretion was measured for 24 h on membrane 1 (PSA array1), (**b**) 48 h on membrane 2 (PSA array2) and (**c**) 72 h on membrane 3 (PSA array 3). Each spot on the membrane represents PSA from a single well. The blue squares highlight a specific area of the membrane at different time intervals, white and yellow arrow indicate PSA from a single well at 24, 48 and 72 h.
